# Linking Heart Health and Mental Wellbeing: Centering Indigenous Perspectives from across Canada

**DOI:** 10.3390/jcm11216485

**Published:** 2022-11-01

**Authors:** Shannon N. Field, Rosalin M. Miles, Darren E. R. Warburton

**Affiliations:** 1 Indigenous Health & Physical Activity Program, Faculty of Education, University of British Columbia, Vancouver, BC V6T 1Z4, Canada; 2 Physical Activity and Chronic Disease Prevention Unit, Faculty of Education, University of British Columbia, Vancouver, BC V6T 1Z4, Canada; 3 Indigenous Physical Activity and Cultural Circle, Vancouver, BC V6N 3S1, Canada

**Keywords:** Indigenous, mental health, heart health, cardiac, wholistic wellness

## Abstract

Indigenous peoples have thrived since time immemorial across North America; however, over the past three to four generations there has been a marked increase in health disparities amongst Indigenous peoples versus the general population. Heart disease and mental health issues have been well documented and appear to be interrelated within Indigenous peoples across Canada. However, Western medicine has yet to clearly identify the reasons for the increased prevalence of heart disease and mental health issues and their relationship. In this narrative review, we discuss how Indigenous perspectives of health and wholistic wellness may provide greater insight into the connection between heart disease and mental wellbeing within Indigenous peoples and communities across Canada. We argue that colonization (and its institutions, such as the Indian Residential School system) and a failure to include or acknowledge traditional Indigenous health and wellness practices and beliefs within Western medicine have accelerated these health disparities within Indigenous peoples. We summarize some of the many Indigenous cultural perspectives and wholistic approaches to heart health and mental wellbeing. Lastly, we provide recommendations that support and wholistic perspective and Indigenous peoples on their journey of heart health and mental wellbeing.

## 1. Introduction

Since time immemorial, Indigenous peoples have learned to thrive across North America, attending to wellness through interconnected wholistic practices. Unfortunately, colonization has disrupted these ways, leading to drastic health disparities between Indigenous peoples and non-Indigenous Canadians. Heart disease and mental health illnesses have been well documented as comorbidities, both of which are experienced by Indigenous peoples at higher rates of morbidity and mortality. Western research has yet to fully elucidate the reasons for the increased rates of heart disease and mental health issues and their relationship. However, Indigenous perspectives of wellness can provide a distinct perspective regarding their connection.

This paper is designed to provide a narrative review of the prevalence of heart disease and mental health issues in Canada. We provide a summary of current literature involving Indigenous perspectives related to health and wholistic wellness with specific reference to the importance of incorporating these perspectives when addressing the burden of heart disease and mental health issues within Indigenous peoples and communities. This paper recommends that all cardiac health care providers consider the interplay of mental health and heart disease, particularly within the context of Indigenous perspectives of heart health and wholistic wellbeing given the prevalence of these health disparities within this population. This review is designed to improve support for Indigenous heart patients, while providing greater insight into wholistic health and wellness. Finally, this paper offers practical recommendations for improving the health system for the betterment of Indigenous health and wellness.

## 2. Positioning the Authors

Positioning or locating the authors/researchers is a common practice within Indigenous research methodologies [1,2]. It is a demonstration of our relations and suggests particular knowledge sets and biases we may hold.

Shannon Field is Métis with roots in the Red River settlement and has mixed European ancestry. She grew up on the unceded traditional territory of the Musqueam, Squamish, and Tsleil-Waututh First Nations, also known as Vancouver, and continues to live there as she completes her Master of Science degree. Her current graduate work is in kinesiology and Indigenous research methods, and she has also worked in the area of Indigenous health, research, and cultural safety for over 5 years. She continues to learn about Indigenous health and wellness under the guidance of Dr. Rosalin Miles and Dr. Darren Warburton among others in the Indigenous Health and Physical Activity program, including her direct graduate supervisor, Dr. Shannon Bredin.

Rosalin Miles belongs to Lytton First Nation and is a Nlaka’pamux woman. She currently resides with the unceded and traditional territory of the Musqueam community. She has completed her Education, Doctoral Degree, Curriculum and Instruction, specializing in Exercise Science. She works at the University of British Columbia as a Research Associate within the Indigenous Health and Physical Activity program.

Darren Warburton is a mixed ancestry scholar who grew up in Treaty 20 Michi Saagiig (“Mississauge”) Territory and in the traditional territory of the Michi Saagiig (“Mississauge”) and Chippewa Nations, collectively known as the Williams Treaties First Nations. He currently works, lives, and plays in the traditional, ancestral, and unceded territories of the Musqueam peoples. His research supports Indigenous self-determination, empowerment, health, and wellbeing through Indigenous led, co-created, and community-based approaches.

## 3. Prevalence of Heart Disease in Canada

Among the top ten chronic conditions experienced by Canadian adults are those relating to poor heart health, including hypertension (25%) and ischemic heart disease (8%) [3]. Across Canada, diseases of the heart are the second leading cause of death, with essential hypertension and hypertensive renal disease, and atherosclerosis among the top 20 [4]. While comprehensive data is not available for Indigenous peoples in Canada, existing data does point toward higher prevalence and growing rates of heart diseases, with data suggesting rates from two to ten times that of other Canadians [5,6,7]. Indigenous peoples also have higher rates of risk factors for heart disease including obesity, diabetes, smoking, physical inactivity, and other cardiovascular disease risk factors [5,7,8]. This increase in prevalence is due to the lasting effects of colonization (and its institutions, such as the Indian residential school system). In recognition of these health disparities, one of the six recommendations in the Canadian Heart Health Strategy and Action Plan (2009), was to address this “crisis” and to ensure that Indigenous peoples have access to the same quality of health services as the rest of Canada [9].

## 4. Prevalence of Mental Health Issues in Canada

Furthermore, in the top ten chronic conditions experienced by Canadian adults, are those relating to poor mental health, including mood and/or anxiety disorders (13%) and dementia (8%) [3]. Mental health issues and/or illnesses are characterized by a range of behaviours, thoughts, and emotions leading to distress or impaired functioning in daily life [10]. Approximately 20% of the Canadian population are living with a mental health problem or illness at any given time [10].

The rates of mental health stability for Indigenous peoples vary from community to community; however, on average the rates of depression within Indigenous peoples are twice that of the Canadian average, and suicide rates are two to 11 times higher than non-Indigenous Canadians [11]. In addition, Indigenous peoples are also subject to higher rates of poverty, unemployment, housing and food insecurity, and social exclusion and discrimination, all of which have impacts to mental health [12,13,14].

## 5. Heart Disease and Mental Illness as Comorbidities

The prevalence of cardiac disease and mental health issues or illnesses as comorbidities has been well documented; however, researchers have yet to fully understand their connection [15]. Many correlational studies have demonstrated this connection; for example, studies have found increased incidences of either heart disease or depression following the diagnosis of either condition [16]. A meta-analysis looking at patients with heart failure found that approximately 20% also had depression [15].

Numerous studies have shown a relationship between anxiety and cardiovascular disease [17]. Anxiety has been shown to increase risk of coronary heart disease independent of demographic variables, biological risk factors, and health behaviours [18]. In a study aimed at understanding Indigenous women’s perspectives on heart health, participants shared that they worried about their heart health, and symptoms like a racing heart exacerbated this fear—not knowing if it was because of anxiety, an acute cardiac episode, or both [19].

Other studies have looked at biomarkers, such as allostatic load and hypothalamic-pituitary-adrenal (HPA) axis activity in relation to heart disease [20,21]. Low socioeconomic status, racism, and adverse childhood experiences, which are common experiences in Indigenous populations [14], are all linked to higher allostatic loads [21]; and higher allostatic load has been linked to various cardiovascular diseases, including ischemic heart disease and hypertension as well as poorer mental health, including depression and anxiety [21]. Numerous Indigenous peoples have cited systemic discrimination and colonization (which are also associated with the above factors) as attributing to their heart disease [22,23].

HPA axis activity has also been linked to heart health [20]. In a longitudinal study with healthy adolescents, psychological health was shown to be an important factor in endothelial function [20]. Anger, depression, and anxiety negatively affected HPA axis activity, while high self-concept had a positive effect [20]. In a large adult population study, positive affect has also been associated with a reduced risk of 10-year incident of cardiovascular disease [24]. While allostatic load and HPA axis activity are characterized by biomarkers, they have yet to become clear distinguishing factors in disease causation [18].

Moreover, little research has been conducted in Canada with First Nations, Métis, and Inuit peoples regarding the connection between cardiac disease and mental health. The high rate of co-occurrence, overlapping risk factors, and the prevalence of these two medical conditions in Indigenous populations warrants a further consideration into how and why these two health issues are interconnected, particularly from the perspectives of Indigenous peoples. Efforts in the prevention and treatment of cardiac disease should consider mental health factors, more wholistic health perspectives (such as emotional and spiritual health), and social, political, and environmental factors [25,26]. Importantly, wholistic perspectives of health have long been held by Indigenous peoples and we have much to learn from their knowledge.

## 6. Why Are Indigenous Peoples More Likely to Have These Conditions?

While the incidence of Indigenous peoples having heart disease and mental health problems is higher than other Canadians, it is important to consider why this is the case. The biomedical model has typically focused on genetics and “lifestyle” choices, resulting in blaming Indigenous peoples for their own poor health [26]. However, social determinants of health, including racism and colonization, can further explain why Indigenous peoples have a higher prevalence of these health conditions as well as the risk factors for their development [25,26].

Colonization has disrupted Indigenous ways of being, including ceremonies, relational systems, and connections to land that supported the wellbeing of individuals and Nations for generations. Activities such as hunting, and gathering food and medicines were interrupted, changing Indigenous peoples’ nutrition, healing, and the traditional physical activities they had access to and participated in daily [22]. Indigenous peoples were segregated and had to become dependent on Government rations, and groceries available in stores that were full of processed and sugary foods [22]. Poor nutritional diets and physical inactivity are major contributing factors to heart disease and mental health [27,28].

Anand et al. found that First Nations communities with reported greater socioeconomic advantage (such as greater employment, income, and long-term marital partnerships), higher proportions of individuals with completed high school education, trust amongst community members, and high social support had a lower burden of cardiovascular risk factors [29]. They also found that those with difficulty accessing primary care and prescription medications increased the burden of risk factors and carotid atherosclerosis [29]. When comparing their results to a parallel study among non-Indigenous Canadians, they found that non-Indigenous people scored lower for risk factors, and fewer people were classified as high risk demonstrating the considerably higher burden of risk factors in First Nations communities [29]. The lack of access to health care and socioeconomic discrimination are some of the legacies of colonization [30].

Other legacies include oppressive systems and institutions which have excluded Indigenous voices and greatly impacted Indigenous peoples’ health and wellness [22,25,26,31,32]. Numerous Indigenous peoples across Canada have attributed their heart disease to stress, grief and inter-generational trauma due to oppressive systems [22,23,33]. The trauma stemming from colonial policies, such as the Indian residential school system that removed children from their families and placed them into far away institutions, has resulted in generations of Indigenous people who suffered severe emotional, mental, physical, and sexual abuse and traumas [26,31,34]. Despite the violence children experienced in residential schools and the grief over the severance of relationships with family members and home communities, most children were forced to suppress emotions, leaving them to tend to their broken hearts in isolation [22,23].

Psychological distress has a strong dose-dependent association with heart attack and stroke even when confounding variables, including sociodemographic factors, lifestyle factors, physiological facts and family history, are controlled for [35]. The First Nations Information Governance Committee, which collects data from First Nation community members across Canada, found that individuals who attended the Indian residential schools have much higher rates of diagnosis for heart disease (8.3%) and hypertension (23.0%) compared to those who had not attended these schools (4.0% and 13.0% respectively) [36]. These statistics demonstrate the detrimental impacts of what these First Nation survivors experienced and the damaging impacts of these colonial institutions.

Needless to say, it is important to understand the historical and cultural context in which illnesses such as heart disease and mental health problems arise. Detrimental social determinants, including racism and discrimination increase the risk burden for such health conditions for Indigenous peoples [14,26,31,32]. Dr. Moneca Sinclaire has “shared that any health study concerning Indigenous people today will likely say less about Indigenous people and more about what happens when you colonize a group of people for centuries” [25] (p. S151). Her quote speaks to the significant impact colonization has had on Indigenous peoples, as well as the fact that Western research is typically focused on health disparities and burden of illness, rather than Indigenous knowledge of health and wellbeing.

## 7. Wholistic Health Models Are Needed to Understand Intersecting Health Conditions

The consideration of the context in which health conditions arise, should include an examination of models of health in use and whether Indigenous voices are being included. How we treat illnesses such as heart disease and mental health problems is dictated by the models of health used, with the biomedical model dominating Western health systems used across Canada for the last century [37]. This model assumes that disease is the single underlying cause of all illness, and that health is the absence of disease. However, due to the inability to explain many relational causes of poor health, including somatic conditions, many health professionals are looking to other progressive models of health. One popular example is the biopsychosocial model, which was proposed in 1977 by George Engel [38]. This model acknowledges the influence of psychological and social factors on a patient’s perception and experience of health [37,38]. The Mental Health Commission of Canada for one, recognizes that mental health problems and illnesses stem from complex interactions between social, economic, psychological, and biological factors [10]; these factors intersect to play a role in other aspects of our overall health and wellbeing.

Indigenous peoples since time immemorial have believed that health centers around balance and that different aspects of wellness are completely interconnected. Again, while each Nation has its own unique cultural teachings, typically this wholistic perspective incorporates four dimensions of wellness, including physical, spiritual, emotional, and mental, and extends beyond the individual to include family, community, and relationships with the land [14,39]. Indigenous perspectives and teachings also focus on strength-based or proactive approaches, which encourages healing and wellness from which ever state a person is in and based on their inherent wholistic gifts [39].

Although more comprehensive health models have been gaining popularity within Western health systems, aspects of Indigenous health models continue to be excluded [32]. Indigenous patients and families also continue to feel unheard, disrespected, and experience blatant racism in the health care system [25,32]. Because of this, numerous Indigenous health care services have been established to offer culturally safer care [40,41]. The inclusion of cultural values, customs and beliefs, and traditional healing practices within these services are central strategies and have produced promising results in patient satisfaction and health outcomes [41,42,43].

## 8. The Link between Heart and Mental Health: Centering Indigenous Perspectives from across Canada

There are over 50 distinct Indigenous Nations, representing over 600 First Nations communities across what is now called Canada [44]. Each Nation has unique languages, ways of being, and cultural teachings regarding personhood, spirituality, and the body. However, amongst this diversity, these Nations typically share common worldviews and ways of being including the integration of wholistic wellness practices throughout all aspects of life. These views often include maintaining balance and connection with oneself, family, and community as it is seen as essential for the wellbeing of Indigenous peoples [14,22,39,45]. This section will further discuss some of the Indigenous cultural views that have been shared publicly regarding heart health and how this often extends to being healthy in mind, body, spirit, and having good relationships with others. Before sharing some interpretations from Indigenous peoples, it is important to consider that the English language can be limiting, lacking the words to fully describe Indigenous conceptions [1]. These are also short quotes and references that may lack context in their teachings. Please continue through this section with an open heart and open mind, and know that these concepts go much deeper than their words.

Many Indigenous peoples believe that the heart has strong cultural meanings, and is often referred to as being the center and the connection of everything [33,46,47]. In some Indigenous cultures, such as the Anishinaabe, the strawberry is a symbolic representation of the heart and is referred to as heart berry; not only because of its shape, but also because of the vast plant system spreading out with leaves, shoots, and roots similar to how our heart uses the circulatory system to connect to all organs in the human body [47]. Moreover, the heart berry also stands as a reminder of the connection between the body, mind, emotions, and spirit, and guides in balance among these [47].

Other Indigenous Nations understand the heart to not only be connected to other body systems, but to operate as a single unit. Musqueam Elder Larry Grant simply articulates that “the heart and mind function as one” [38]. When asked, Pnnal Jerome in Gesgapegiag described that there is no word for heart in Mi’kmawi’simk (Mi’gmaq language) but rather a word that described the whole person, because in Mi’gmaq culture, the heart, mind, and spirit are not separate [46]. A Cree woman is quoted saying that the heart “is gifted to every human being at birth by the Creator and is where one’s emotions and intelligence are derived” [22]. These quotes demonstrate the strong connections between the heart and the mind within varying Indigenous cultures across Canada.

Like the previous quotes, participant Esther Sanderson from Opaskwayak Cree Nation (the Pas, Manitoba) shared a reflection on the meaning of her heart recognizing that it has two functions:

“*It is a physical organ that pumps blood through my body, and second is the blood flowing into my heart that carries my ancestors’ Cree language, ceremonies, songs, values and life teachings. It is the same blood that has flowed through my ancestors that flows through me*”.

[48]

Sanderson’s quote also illustrates the understanding of deep generational ties and our relations’ contribution to wholistic heart health and personal wellness.

What she describes is often referred to as ‘blood memory’. Blood memory is the ability to pass down ancestral knowledge, wisdom, and history [49,50]. It extends beyond genetic inheritance of disease to inheritance of all that makes us well [49]. This also relates to the seven generations principle. Numerous Indigenous peoples, including Cree First Nations, believe that our actions influence life for the next seven generations, and therefore we must act responsibly in consideration of the future of our families and communities [51].

This includes the consideration of our heart health and how we take care of one another. Eliza Beardy, who is Oji Cree from Wasagamack First Nation, Manitoba, has emphasized that in order for our children to be healthy, we need to be healthy and heal our hearts [52]. Not only do we have impact on future generations, but future generations can make us well too. Virginia McKay who is Saultaux and living in Berens River First Nation, Manitoba, recognizes her grandchildren as a source of healing for her [53]. In a family interview, one man’s daughters also shared how they cared for one another during their grandfather’s struggle with heart disease, making sure everyone had good food to eat and the social support they needed [54]. They also went to ceremony and prayed for their family members after their grandfather had passed away [54]. Their father also describes “broken heart syndrome” which happens when someone with few relationships dies following the death of a loved one. However, when there are enough relations around, such as children and grandchildren, then the heart can heal itself and this person can go on to process grief and live a healthy life [54]. The quotes from these Indigenous peoples discussing heart health eloquently expresses the wholistic view of how the body’s physical health is intertwined with mental, emotional, and spiritual wellbeing, and family and community.

## 9. Improving Heart Health and Mental Health through Wholistic Approaches

The use of wholistic and Indigenous approaches to wellness are recommended to improve the health outcomes for both heart and mental health patients. Recognizing that they are commonly existing comorbidities, efforts towards wholistic wellbeing may be beneficial in prevention and treatment. Cultural approaches within service delivery and as a form of ‘treatment’ are increasingly being recommended and employed by Indigenous communities to repair the trauma of colonialism, oppressive systems, and promote wholistic well-being amongst its communities [40,43,55]. These recommendations include creating opportunities to strengthen cultural identity, participate in cultural and traditional activities, and developing strong connections with family and community.

A strong sense of cultural identity is a valuable contributor to mental health and overall wellbeing [13,56,57]. In a study exploring a culturally responsive, land-based initiative on the mental health of Indigenous youth, Walker et al. heard that students learned about their identity from their family, through language, and through school [58]. Furthermore, that the initiatives, which offered numerous cultural activities specific to their culture, community, geography, and language, changed the way the youth felt about themselves, describing that afterwards they felt good with one student quote as saying “I do feel a change like I feel that my spirit is fed like with goodness” [58]. Snowshoe et al. [59] found that cultural connectedness including knowledge and association with identity, traditions and spiritual practices, was a positive contributor to mental wellbeing.

First Nations and Métis people have found that activities, such as traditional arts, songs, dance, and ceremonies, were beneficial to their overall wholistic wellbeing [56,58]. Indigenous youth who participated in a land-based program said that participating in cultural activities, like attending ceremony, beading, and listening to pow wow music benefitted their mental health through grounding and reducing stress [58]. While we have yet to find literature on the direct connection between cultural connectedness and reductions in heart disease, the discussed improvements in mental health are likely to also be associated with positive associations for cardiac health.

Additionally, having access to traditional land is important for hunting and gathering food and medicines. In turn, this elicits revitalization of storytelling, sharing of teachings, physical activities, healthy eating, and access to traditional medicines that have kept Indigenous peoples well since time immemorial. There is a substantial amount of literature about the benefits of practicing physical activity and healthy eating for both heart health and mental health [60,61,62].

However, cultural and traditional activities are not the only ways in which Indigenous peoples tend to their wellness. First Nations adults and youth noted that (starting from the highest mode) sleep, diet, happiness, good social supports, reduced stress, and physical activity contributed to their physical, emotional, mental, and spiritual health [63]. One study also found that a number of Indigenous women diagnosed with a heart condition also turned to spiritual health practices (including traditional Indigenous cultural practices as well as religious practices from Christianity) as a way to treat and heal from their condition [19]. Indigenous mental health counsellors spoke to their wholistic approaches citing that they also discussed with their clients the inclusion of cultural practices as well as health care topics like seeing a medical doctor, diet, and sleep [57].

Indigenous perspectives of health and wellbeing also extend to include family and community supports [14]. Many Indigenous peoples cite attending family gatherings and going to ceremony are ways to improve heart health [22,33]. Some Indigenous women spoke about their relationship with their grandchildren, and how they found them as a vital component for their healing [22]. Further to this, many women wanted to be healthy for their grandchildren and mentor them on healthy habits [22]. In many Indigenous cultures there is a great responsibility and importance placed on caring for future generations [51]. Many Indigenous peoples also believe that the overall health and wellbeing of their community and social networks are important contributing factors to their mental health wellbeing [56,57]. First Nations and Métis participants named social and cultural activities as places to build respectful relationships and contribute to supporting one another, providing a sense of fulfillment [56]. For many Indigenous peoples, their community is seen as an extension of their individual wellbeing and it is seen as a necessary contributing factor in wholistic balance and wellness [14,56,57].

## 10. Recommendations for Improving Cardiac Care for Indigenous Patients

As we come to the end of this article, we would like to leave you with several recommendations for improving cardiac care for Indigenous patients. For one, a level of system change is needed to allow for Indigenous knowledges, perspectives, and physical bodies to safely enter the health system [32]. While this may feel out of reach, systems are made up of people, and therefore it is individual people who change the system. In acknowledgement that everyone and anyone can contribute to the vision for a culturally safe health care system, the First Nations Health Authority in British Columbia started the “it starts with me” campaign [64]. As health professionals, you have the power to advocate for and create change to improve health care for Indigenous peoples.

In line with the concept of “it starts with me”, progress towards culturally safe health care requires a practice of self-reflection including understanding personal and systemic conditioned biases [64,65]. Understanding cultural differences can be helpful, but the countless cultural practices and variance in peoples’ knowledge and practice of their own culture is too great to rely on this knowledge and can lead to poor judgements [65]. Part of culturally safe practice is humility—the ability to know and accept that we do not always know the answers or the fact that we could be wrong [65]. Cardiac health care providers will have to relinquish their expert title, recognizing their patient as the expert in their own life. Only the patient is truly able to identify and explain the impacts of their identity and experiences on their wellbeing [65]. The practice of humility is brought forth through continuous self-reflection, bias-minding, and deep listening and learning from others who have different perspectives and experiences than you (including your patients).

One of the other key tenets of cultural safety is understanding the history of Canada, the historical and present-day impacts of colonization, and Indigenous knowledges of this history. Within this article, you may have come to the understanding that the health disparities between Indigenous peoples and non-Indigenous Canadians are greatly influenced by colonization, systemic oppression, and racism. Understanding this context helps us by situating our patients in broader social and political powers that may have had greater influence on the development of their heart disease than their individual behaviours. In combination with the practice of self-reflection and humility, we can dismantle any stereotypes, assumptions, and blame that are often applied to Indigenous patients and begin focusing on the quality health care they deserve.

Without this practice of humility, health care providers often make well-intended treatment suggestions including lifestyle advice, such as diet changes and increasing physical activity, without realizing the difficulty some patients may have in achieving these things. Medved et al. [19] spoke with Indigenous women who felt like they were being controlled and dismissed by their health care providers. It reminded them of times when they were highly controlled in the Indian residential school system, and the advice they were given regarding their heart health was irrelevant to them given their competing priorities and lack of access to the recommended resources.

Consequently, taking the time to listen and learn from your Indigenous heart patients can go a long way. This active form of listening with care can be considered patient-centered care, which encompasses relationship building, attentiveness, and empowering the patient with autonomy in their care decisions and planning [66]. Lee and Lin [66] found that patients who perceived greater autonomy had higher trust and satisfaction with their physicians, and that autonomy support was beneficial for mental health, and has the potential to benefit physical health. In discussion around care planning, let your patient communicate their priorities and determine what course of action will suite their lifestyle. Collaborating with your patients and their families will provide better insights on creating care plans that are meaningful and achievable.

We also recommend asking the patient about how family and community might be involved in their wellbeing. As discussed earlier in this article, family and community are important parts of wellbeing [14,39]. Ask the patient about who supports them and encourage them to continue to surround themselves with family if the patient indicates they would like their involvement. Including family in care planning conversations means that they can contribute to the healing of their family member at home.

Have conversations with your patients about their beliefs around heart health and mental wellbeing. Listening to Indigenous patients can also further your knowledge of Indigenous cultures and wholistic health perspectives but be mindful that not all Indigenous peoples carry or follow traditional knowledge of wellness, nor are they obligated to share that knowledge with you. Colonial ideologies have left no room for Indigenous knowledge, healing, or medicines in the Western health care system [22,32]. Though, in the journey towards reconciliation, many Canadian hospitals are taking on Indigenous health programs and seeing positive outcomes [67,68,69]. Therefore, if you patient does share knowledge of traditional practices, encourage them to continue doing so and incorporate it into the care plan. If you work in a large health center, find out if there is an Indigenous health team that can further support Indigenous patients on their journey to a healthy heart.

Incorporating culture into health care planning may achieve better health outcomes and improve wellbeing [39,56]. Access to culture could be a promising way to improve mental health and physical health conditions such as heart disease; however, regaining connection to culture may be a difficult journey, for example, if the patient does not live close to their community or has lost ties to family due to being placed in foster care. Medved et al. [19], noted that cultural practices of healing “demands a comprehensive commitment to a way of inhabiting the world that the women said was no longer accessible to them” (p. 1620). Some participants in their study were even astonished when the researchers introduced the topic of incorporating Indigenous healing practices. Medved et al. [19] attribute this shock to the uptake of Western healing practices and forced distancing of cultural practices throughout colonization. Again, health care providers should work with patients to discuss their aspirations and what works best for them, as this may or may not include cultural aspects or ties to community.

Finally, always used a strengths-based approach. Instead of focusing on what not to do, focus on what patients can do. For example, encourage them to do things that make them happy and reduce stress rather than focusing on the avoidance of stress-inducing things. Choose things together with your patient that they can work towards that will benefit their heart health.

Working towards improving cardiac care for Indigenous patients should include learning and understanding the context in which health disparities exist, understanding Indigenous perspectives of health and wellness, and creating deeper relationships with Indigenous patients to understand their aspirations and needs. In order to reduce health disparities for Indigenous peoples, including in heart health and mental health, it is imperative that Indigenous peoples, knowledges, and ways of being are included in current health models as legitimate approaches and valued health and wellness practices.

## 11. Conclusions

While Indigenous peoples may endure higher rates of heart disease and mental health issues, it is important to understand how the impacts of colonization have created these disparities and exclusion of Indigenous perspectives and practices within today’s health care system. Being open to Indigenous knowledge and wholistic perspectives of wellness would improve health care staff’s understanding as to how certain illnesses may arise at higher rates for Indigenous patients, such as that as discussed in this paper.

Upon dismantling racial and oppressive barriers and working alongside Indigenous peoples, we can create a health care system that provides better health and wellness models for Indigenous peoples that allows them to exercise their right to self-determination. In turn, this will lead to improved wholistic health and wellness outcomes, reduce the existing health disparities, and improve the health care for all patients as a result of using strength-based approaches with family and community.

## Data Availability

Not applicable.

## References

[1] Kovach M. (2009). Indigenous Methodologies: Characteristics, Conversations, and Contexts.

[2] Wilson S. (2008). Research Is Ceremony: Indigenous Research Methods.

[3] Public Health Agency of Canada Prevalence of Chronic Diseases among Canadian Adults. https://www.canada.ca/en/public-health/services/chronic-diseases/prevalence-canadian-adults-infographic-2019.html.

[4] Statistics Canada Table 13-10-0394-01 Leading Causes of Death, Total Population, by Age Group. https://www150.statcan.gc.ca/t1/tbl1/en/tv.action?pid=1310039401.

[5] Anand S.S., Yusuf S., Jacobs R., Davis A.D., Yi Q., Gerstein H., Montague P.A., Lonn E. (2001). Risk Factors, Atherosclerosis, and Cardiovascular Disease among Aboriginal People in Canada: The Study of Health Assessment and Risk Evaluation in Aboriginal Peoples (SHARE-AP). Lancet.

[6] Reading J. (2015). Confronting the Growing Crisis of Cardiovascular Disease and Heart Health Among Aboriginal Peoples in Canada. Can. J. Cardiol..

[7] Reading J. (2009). The Crisis of Chronic Disease among Aboriginal Peoples: A Challenge for Public Health, Population Health and Social Policy.

[8] Foulds H.J.A., Bredin S.S.D., Warburton D.E.R. (2011). The Effectiveness of Community Based Physical Activity Interventions with Aboriginal Peoples. Prev. Med..

[9] Smith E.R. (2009). The Canadian Heart Health Strategy and Action Plan. Can. J. Cardiol..

[10] Mental Health Commission of Canada (2013). Making the Case for Investing in Mental Health in Canada.

[11] Khan S. (2008). Aboriginal mental health the statistical reality. Visions.

[12] Boksa P., Joober R., Kirmayer L.J. (2015). Mental Wellness in Canada’s Aboriginal Communities: Striving toward Reconciliation. J. Psychiatry Neurosci..

[13] Williams A.D., Clark T.C., Lewycka S. (2018). The Associations Between Cultural Identity and Mental Health Outcomes for Indigenous M ¯ Aori Youth in New Zealand. Front. Public Health.

[14] Reading C.L., Wien F. (2009). Health Inequalities and Social Determinants of Aboriginal Peoples’ Health.

[15] Rutledge T., Reis V.A., Linke S.E., Greenberg B.H., Mills P.J. (2006). Depression in Heart Failure a Meta-Analytic Review of Prevalence, Intervention Effects, and Associations with Clinical Outcomes. J. Am. Coll. Cardiol..

[16] Mosovich S.A., Boone R.T., Reichenberg A., Bansilal S., Shaffer J., Dahlman K., Harvey P.D., Farkouh M.E. (2008). New Insights into the Link between Cardiovascular Disease and Depression. Int. J. Clin. Pract..

[17] Harlapur M., Shimbo D. (2020). Anxiety and Heart Disease. Encyclopedia of Behavioral Medicine.

[18] Roest A.M., Martens E.J., de Jonge P., Denollet J. (2010). Anxiety and Risk of Incident Coronary Heart Disease. A Meta-Analysis. J. Am. Coll. Cardiol..

[19] Medved M.I., Brockmeier J., Morach J., Chartier-Courchene L. (2013). Broken Heart Stories: Understanding Aboriginal Women’s Cardiac Problems. Qual. Health Res..

[20] Chen Y., Osika W., Dangardt F., Friberg P. (2017). Impact of Psychological Health on Peripheral Endothelial Function and the HPA-Axis Activity in Healthy Adolescents. Atherosclerosis.

[21] Guidi J., Lucente M., Sonino N., Fava G.A. (2021). Standard Review Article Allostatic Load and Its Impact on Health: A Systematic Review. Psychother. Psychosom..

[22] Fontaine L.S., Wood S., Forbes L., Schultz A.S.H. (2019). Listening to First Nations Women’ Expressions of Heart Health: Mite Achimowin Digital Storytelling Study. Int. J. Circumpolar Health.

[23] Conklin A., Lee R., Reading J., Humphries K. (2019). Promoting Indigenous Women’s Heart Health: Lessons from Gatherings with Elders and Knowledge-Holders.

[24] Davidson K.W., Mostofsky E., Whang W. (2010). Don’t Worry, Be Happy: Positive Affect and Reduced 10-Year Incident Coronary Heart Disease: The Canadian Nova Scotia Health Survey. Eur. Heart J..

[25] Schultz A., Nguyen T., Sinclaire M., Fransoo R., McGibbon E. (2021). Historical and Continued Colonial Impacts on Heart Health of Indigenous Peoples in Canada: What’s Reconciliation Got to Do with It?. CJC Open.

[26] Mcgibbon E., Waldron I., Bscn J.J. (2013). The Social Determinants of Cardiovascular Disease: Time for a Focus on Racism. Divers. Equal. Health Care.

[27] Parletta N., Milte C.M., Meyer B.J. (2013). Nutritional Modulation of Cognitive Function and Mental Health. J. Nutr. Biochem..

[28] Ravera A., Carubelli V., Sciatti E., Bonadei I., Gorga E., Cani D., Vizzardi E., Metra M., Lombardi C. (2016). Nutrition and Cardiovascular Disease: Finding the Perfect Recipe for Cardiovascular Health. Nutrients.

[29] Anand S.S., Abonyi S., Arbour L., Balasubramanian K., Brook J., Castleden H., Chrisjohn V., Cornelius I., Davis A.D., Desai D. (2019). Explaining the Variability in Cardiovascular Risk Factors among First Nations Communities in Canada: A Population-Based Study. Lancet Planet. Health.

[30] Adelson N. (2005). The Embodiment of Inequity: Health Disparities in Aboriginal Canada. Can. J. Public Health.

[31] Wesley-Esquimaux C.C., Smolewski M. (2004). Historic Trauma and Aboriginal Healing.

[32] Turpel-Lafond M.E. (2020). In Plain Sight: Addressing Indigenous-Specific Racism and Discrimination in BC Health Care Summary Report.

[33] Sinclaire M. Documentary Two: “Indigenous and Biomedical Ways to Treat the Heart”. http://www.sbrc.ca/schultz/.

[34] National Centre for Truth and Reconciliation Residential School History. https://nctr.ca/education/teaching-resources/residential-school-history/.

[35] Jackson C.A., Sudlow C.L.M., Mishra G.D. (2018). Psychological Distress and Risk of Myocardial Infarction and Stroke in the 45 and Up Study. Circ. Cardiovasc. Qual. Outcomes.

[36] First Nations Information Governance Centre (2007). First Nations Regional Longitudinal Health Survey (RHS) 2002/03: Results for Adults, Youth and Children Living in First Nations Communities.

[37] Wade D.T., Halligan P.W. (2004). Do Biomedical Models of Illness Make for Good Healthcare Systems?. BMJ.

[38] Bolton D., Gillett G. (2019). The Biopsychosocial Model of Health and Disease New Philosophical and Scientific Developments.

[39] Howell T., Auger M., Gomes T., Brown F.L., Young Leon A. (2016). Sharing Our Wisdom: A Holistic Aboriginal Health Initiative. Int. J. Indig. Health.

[40] Harfield S.G., Davy C., McArthur A., Munn Z., Brown A., Brown N. (2018). Characteristics of Indigenous Primary Health Care Service Delivery Models: A Systematic Scoping Review. Glob. Health.

[41] Mccalman J., Heyeres M., Campbell S., Bainbridge R., Chamberlain C., Strobel N., Ruben A. (2017). Family-Centred Interventions by Primary Healthcare Services for Indigenous Early Childhood Wellbeing in Australia, Canada, New Zealand and the United States: A Systematic Scoping Review. BMC Pregnancy Childbirth.

[42] Lai H., Miles R., Bredin S., Kaufman K., Chua C., Hare J., Norman M.E., Rhodes R.E., Oh P., Warburton D.E.R. (2019). “With Every Step, We Grow Stronger”: The Cardiometabolic Benefits of an Indigenous-Led and Community-Based Healthy Lifestyle Intervention. J. Clin. Med..

[43] Hayward M.N., Pace R., Zaran H., Dyck R., Hanley A.J., Green M.E., Bhattacharyya O., Zwarenstein M., Emond J., Benoit C. (2020). Closing the Indigenous Health Gap in Canada: Results from the TransFORmation of IndiGEnous PrimAry HEAlthcare Delivery (FORGE AHEAD) Program. Diabetes Res. Clin. Pract..

[44] Indigenous Peoples and Communities. https://www.rcaanc-cirnac.gc.ca/eng/1100100013785/1529102490303.

[45] National Collaborating Centre for Indigenous Health Video: Mite Achimowin-Heart Talk Introduction. https://www.nccih.ca/495/Video__mite_achimowin_-_Heart_Talk_Introduction.nccih?id=282.

[46] Nadeau D.M. (2020). Unsettling Spirit: A Journey into Decolonization.

[47] Wabano Centre Strawberry Teachings. https://wabano.com/product/strawberry-teachings/.

[48] National Collaborating Centre for Indigenous Health (2017). Episode 4-“MITE MEKIWIN-Gift of the Heart” by Esther Sanderson.

[49] Pember M.A. Blood Memory. https://dailyyonder.com/blood-memory/2010/07/16/.

[50] Mithlo N.M. (2011). Blood Memory and the Arts: Indigenous Genealogies and Imagined Truths. Am. Indian Cult. Res. J..

[51] Makokis J.A., Wahpimaskwasis (Little White Bear) (2008). Nehiyaw Iskwew Kiskinowâtasinahikewina-Paminisowin Namôya Tipeyimisowin: Learning Self Determination through the Sacred. Can. Woman Stud..

[52] National Collaborating Centre for Indigenous Health (2017). Episode 2-“My Heartbeat” by Eliza Beardy.

[53] National Collaborating Centre for Indigenous Health (2017). Episode 3-“NIIN INTEPACHIMOWIN-My Heart Story” by Virginia Mckay.

[54] Sinclaire M. Documentary One: ‘Three Generations and Heart”. http://www.sbrc.ca/schultz/.

[55] Barker B., Goodman A., DeBeck K. (2017). Reclaiming Indigenous Identities: Culture as Strength against Suicide among Indigenous Youth in Canada. Can. J. Public Health.

[56] Iwasaki Y., Bartlett J.G., Gottlieb B., Hall D. (2009). Leisure-Like Pursuits as an Expression of Aboriginal Cultural Strengths and Living Actions. Leis. Sci..

[57] Stewart S.L. (2008). Promoting Indigenous Mental Health: Cultural Perspectives on Healing from Native Counsellors in Canada. Int. J. Health Promot. Educ..

[58] Walker S., Kannan P., Bhawra J., Bhawra J., Foulds H.J.A., Katapally T.R. (2021). The Impact of Culture, Identity and Intergenerational Connections on Indigenous Youth Mental Health: Qualitative Findings from a Longitudinal Digital Health Community Trial. Res. Sq..

[59] Snowshoe A., Crooks C.V., Tremblay P.F., Hinson R.E. (2017). Cultural Connectedness and Its Relation to Mental Wellness for First Nations Youth. J. Prim. Prev..

[60] Warburton D.E.R., Bredin S.S.D. (2017). Health Benefits of Physical Activity: A Systematic Review of Current Systematic Reviews. Curr. Opin. Cardiol..

[61] Tappia P.S., Blewett H. (2020). Nutrition and Cardiovascular Health. Int. J. Mol. Sci..

[62] Adan R.A.H., van der Beek E.M., Buitelaar J.K., Cryan J.F., Hebebrand J., Higgs S., Schellekens H., Dickson S.L. (2019). Nutritional Psychiatry: Towards Improving Mental Health by What You Eat. Eur. Neuropsychopharmacol..

[63] First Nations Information Governance Centre (2018). National Report of the First Nations Regional Health Survey Phase 3: Volume Two.

[64] First Nations Health Authority (2015). #Itstartswithme FNHA’s Policy Statement on Cultural Safety and Humility.

[65] Tervalon M., Murray-Garcia J. (1998). Cultural Humility versus Cultural Competence: A Critical Distinction in Defining Physician Training Outcomes in Multicultural Education. J. Health Care Poor Underserved.

[66] Lee Y.-Y., Lin J.L. (2010). Do Patient Autonomy Preferences Matter? Linking Patient-Centered Care to Patient-physician Relationships and Health Outcomes. Soc. Sci. Med..

[67] Greenwood M., Lindsay N., King J., Loewen D. (2017). Ethical Spaces and Places: Indigenous Cultural Safety in British Columbia Health Care. AlterNative Int. J. Indig. Peoples.

[68] Tu D., Hadjipavlou G., Dehoney J., Price E.R., Dusdal C., Browne A.J., Varcoe C. (2019). Partnering with Indigenous Elders in Primary Care Improves Mental Health Outcomes of Inner-City Indigenous Patients: Prospective Cohort Study. Can. Fam. Physician.

[69] Hadjipavlou G., Varcoe C., Browne A.J., Tu D., Dehoney J., Price R. (2018). “All My Relations”: Experiences and Perceptions of Indigenous Patients Connecting with Indigenous Elders in an Inner City Primary Care Partnership for Mental Health and Well-Being. Can. Med. Assoc. J..

